# Patient Prioritization for Proton Beam Therapy in a Cost-neutral Payer Environment: Use of the Clinical Benefit Score for Resource Allocation

**DOI:** 10.7759/cureus.5703

**Published:** 2019-09-19

**Authors:** Ankur M Sharma, Rahul Khairnar, Emily S Kowalski, Jill Remick, Elizabeth M Nichols, Pranshu Mohindra, Torunn Yock, William Regine, Mark V Mishra

**Affiliations:** 1 Radiation Oncology, University of Maryland School of Medicine, Baltimore, USA; 2 Pharmaceutical Health Services Research, University of Maryland, Baltimore, USA; 3 Radiation Oncology, Massachusetts General Hospital, Boston, USA

**Keywords:** proton therapy, radiotherapy, resource allocation, clinical score, radiation

## Abstract

Objectives

There has been a rapid increase in the number of one- and two-room proton beam therapy (PBT) centers, which may be limited in the number of patients they can treat. The objective of this study was to analyze the impact of the ‘clinical benefit score’ (CBS), utilized as a method for treatment prioritization for PBT operating in a ‘cost-neutral’ proton-photon payer environment.

Materials & methods

This study includes patients considered for PBT at a center that initially had only one or two treatment rooms available for clinical use. Patients were prospectively scored using the CBS, and higher scores were prioritized. The outcome was receipt of PBT and the independent variable was CBS. Crude and adjusted analyses were performed using logistic regression.

Results

There were 2163 patients evaluated. A total of 205 patients (9.5%) were deemed candidates for PBT, which was received by 122 (5.6%) patients. In patients considered for PBT, the mean CBS was 18.7. Patients who were <21 years old, female, non-Caucasian, receiving re-irradiation, and those with Medicare had a higher CBS.

Multivariate analysis adjusting for insurance status revealed both CBS and insurance to be significant predictors for receiving PBT. A unit increase in CBS was associated with 1.04 times increased odds of receiving PBT (OR=1.04, 95%CI: 1.01-1.07, p=0.0145) and having Medicare was associated with 3.13 times increased odds of receiving PBT (OR=3.13, 95%CI: 1.57-6.26, p=0.0012). Subgroup analysis, which only included patients enrolled prior to opening the second gantry, showed 1.05 times increased odds of receiving PBT per unit increase in CBS (OR=1.05, 95%CI: 1.00-1.10, p=0.03) and 2.87 times increased odds of receiving PBT in patients with Medicare (OR=2.87, 95%CI: 1.04-7.92, p=0.04).

Conclusion

The CBS utilized was significantly associated with the receipt of PBT in a cost-neutral payer setting. Physicians may consider the use of CBS as a resource allocation tool.

## Introduction

The use of proton beam therapy (PBT) to treat cancer is rapidly increasing in the United States and throughout the world. There are currently 31 operational PBT facilities in the US, with 13 others under construction or in planning stages. In addition, 49 other PBT facilities are currently operating worldwide, with 48 more under construction or in planning stages [1].

The increased use of PBT can be attributed to the dosimetric benefit when compared to more conventional radiotherapy techniques. Unlike photon radiotherapy, which can deposit high radiation doses in normal tissues in an attempt to cover the target, PBT has the ability to spare these same normal tissues and decrease the integral dose because the dose is deposited as per the “Bragg Peak” to a specified depth, with a very rapid dose fall-off thereafter. This may lead to a significant decrease in radiation-related toxicity and the ability to dose escalate, which has been associated with improved outcomes in certain tumor sites [2-8].

Although the number of PBT facilities is increasing around the world, the recent trend has been towards building smaller centers. In the US, 11 (35%) of the 31 operational facilities are one- or two-room treatment centers; 10 (90%) of those have been opened in the last five years. Of the 13 PBT facilities in the construction or planning phase, 10 (77%) will be one or two gantry centers. This trend has also been observed internationally where 25 (51%) PBT centers in operation are one- or two-room treatment facilities, with 16 (64%) opening within the last five years. Twenty-three (48%) of the 48 facilities that are currently under construction or in the planning phase will be one or two treatment room centers [1].

With the increasing demand for PBT from both patients and physicians, these smaller centers will likely face issues of resource allocation, which will require a prioritization strategy. Previous attempts to identify predictive factors have shown that Medicare status is strongly associated with the receipt of PBT [9]. This finding suggests that the receipt of PBT therapy may be driven by health insurance status rather than clinical factors, which is intuitive, as the cost of PBT to the payer is two to three times the cost of photon radiotherapy [10].

However, in a ‘cost-neutral’ setting, where charges for PBT are similar to those of hospital-based intensity-modulated radiation therapy (IMRT), we hypothesize that non-financial clinical factors will be associated with the receipt of PBT. The objective of this study is to analyze the impact of a clinical scoring algorithm, termed the ‘clinical benefit score’ (CBS), which was utilized as a method for patient prioritization for PBT at a National Cancer Institute (NCI)-designated comprehensive-cancer center operating in a ‘cost-neutral’ proton-photon payer environment.

## Materials and methods

This study included patients who were considered for receipt of PBT at our proton treatment center (PC) from January 6, 2015, to July 18, 2016. The PC is associated with an academic medical center radiation oncology network (AMC-RO), which consists of four photon treatment centers and one PBT facility. The PC is a five gantry (including one fixed beam) facility, which exclusively utilizes pencil beam scanning technology. The PC first began delivering PBT on February 23, 2016, at which time, only one treatment gantry was available for clinical use, with a second gantry opening on April 29, 2016.

Prior to the opening of the PC, it was anticipated that in the early days of operation, demand for PBT treatment slots would exceed treatment availability. Therefore, CBS was developed as a tool to study appropriate resource allocation. This tool was effectively utilized up until the opening of a third treatment gantry on July 19, 2016, after which treatment slots for PBT were more widely available. For this reason, only patients considered for PBT before July 19, 2016, were included in this analysis.

CBS was developed by a panel of proton- and photon- radiation oncologists within the AMC-RO network in order to identify those patients who were more likely to benefit from PBT. The previously published “Proton Priority System” (PROPS) created by Bekelman et al. was used as a starting point for designing the CBS [9]. The specific scoring methodology was then decided upon based on local expert opinion and best clinical practices at the time, which were based on available scientific literature [4-5,8]. The CBS was reviewed and approved by the department chairman prior to its implementation.

The components of the score include the primary disease site being treated, radiation retreatment status (yes or no), age group, enrollment onto a clinical trial or registry (yes or no), the complexity of treatment planning considerations, and patient-specific risk factors for radiation-related complications. The detailed scoring rubric can be seen in Table 1. The score ranges from 0 to 65, and a higher score represents those patients who are more likely to benefit from PBT.

**Table 1:** Detailed scoring rubric for the Clinical Benefit Score (CBS) Abbreviation: GI = gastro-intestinal; CW = chest wall; LNs = lymph nodes; IMNs = internal mammary chain lymph nodes; GU = genitourinary; PA = posterior to anterior beam arrangement; SV = seminal vesical; CNS = central nervous system; GBM = glioblastoma multiforme; OAR = organ at risk; RT = radiotherapy; SLE = systemic lupus erythematosus; IBD = irritable bowel disease; tx = treatment; HIV = human immunodeficiency virus; MD = medical doctor; GE = gastroesophageal 1 Patient is assigned to a single category if they qualify for more than one. Note that only a single point value can be assigned per category/column. 2 If OAR constraint is not part of local practice guideline, can use NRG dose constraint. 3 If there is only one dose level, use the secondary dose constraint category (not primary dose constraint). 4 Non-local practice guideline OAR or NRG dose constraints (ex. Hippocampus (+3)).

Category		Category Points	Patient Points^1^
Primary Disease Site Being Treated			
Head and Neck			
Nasopharynx		10	
Sino-nasal		10	
Oral Cavity		5	
Salivary Gland		5	
Oropharynx		5	
Hypopharynx		5	
Cervical/Supraclavicular Only		5	
Larynx		0	
Thyroid		0	
Esophagus			
Thoracic		10	
GE Junction		10	
Cervical		5	
GI			
Hepato-biliary		10	
Anal		7	
Gastric/Duodenum/Pancreas		5	
Rectal		5	
Thoracic			
Parietal Pleura/Vertebral Body Invasion		10	
Mediastinum (including post-operative lung)		10	
Thymus		10	
Lung Parenchyma		5	
Breast			
LEFT Breast/CW + Regional LNs (including IMNs)		10	
LEFT Breast/CW + Regional LNs (excluding IMNs)		5	
LEFT Whole Breast/CW Only		3	
RIGHT Breast/CW + Regional LNs (including IMNs)		5	
RIGHT Breast/CW + Regional LNs (excluding IMNs)		3	
RIGHT Whole Breast/CW Only		0	
Partial Breast		0	
Gynecologic			
Extended Field Endometrial/Cervix		10	
Vulva (Definitive)/Distal Vagina		7	
Vulva (Post-operative)		5	
Endometrial/Post-op Cervix		5	
Intact Cervix/Proximal Vagina		5	
Ovarian		0	
Male-GU			
Testis (PA/Dog Leg)		7	
Prostate + Whole Pelvic		5	
Prostate/SV Only		2	
Bladder		0	
CNS			
Base of skull/Benign/Low-Grade		10	
Brain, Non-GBM		5	
Brain, GBM		0	
Other			
Retroperitoneum		10	
Bone/Soft tissue/Skin		0	
Retreatment of Same/Overlapping Site			
Yes		10	
No		0	
Age Group			
<21		15	
22-39		10	
40-49		5	
50-59		3	
60+		0	
Clinical Trial (only if patient on study)			
Proton vs. Photon Randomized		10	
Non-randomized, Interventional Study		6	
Registry Study		3	
Off-protocol		0	
Treatment Planning Considerations			
Practice Guidelines OAR constraints exceeeded^2^			
Primary		5	
Secondary^3^		10	
Other^4^		Variable	
OAR Differences Would Make It Unsafe to Treat Patient with Photons		+5	
Patient Risk Factors for RT Complications			
SLE/Scleroderma		5	
IBD (only if relevant to tx)		5	
Other Known Genetic Risk Factors		5	
HIV		3	
Miscellaneous (Requires Review by Chairman and Additional MD)	

The patients most likely to benefit from PBT were identified by the consensus opinion of attending physicians after a thorough presentation and discussion of each case at weekly case conferences. The CBS was then applied to the pre-identified patients by a panel consisting of two radiation oncologists and one radiation therapist. The panel provided consistent application of the CBS and reduced inter-observer variability. Patients with a higher CBS were prioritized for treatment but were only scheduled after obtaining insurance approval for PBT (or willingness to self-pay), so not all patients who were identified as being more likely to benefit from PBT actually received treatment.

The primary outcome in this study was the receipt of PBT in those patients identified as being more likely to benefit, which was defined as a dichotomous variable. The primary exposure in this study was CBS, which was defined as a continuous variable. Using logistic regression, multivariate analysis was performed, which was adjusted for Medicare insurance status, as this covariate has previously been shown to be associated with receipt of PBT [9]. Other than the CBS variables listed above and Medicare insurance status, data collected included demographic information such as gender and race.

A subgroup multivariate analysis was also performed, which only included patients assessed prior to the opening of the second gantry on April 29, 2016. Statistical analyses were performed using SAS 9.4 (SAS Institute, Cary NC, US). The study was approved by the local institutional review board before data collection and statistical analysis.

## Results

Over the study period from January 6, 2015, to July 19, 2016, 2163 patients were evaluated for radiotherapy within the AMC-RO network. There were 205 (9.5%) patients identified as potential candidates to receive PBT based on expert opinion. Of these, 122 (5.6%) patients went on to receive PBT.

Of the 205 patients who were deemed potential candidates to receive PBT, approximately 70% had a primary diagnosis of prostate, breast, or central nervous system (CNS) malignancy. The mean CBS was 18.7 (range 2 - 38, SD 10.7). Those patients who received PBT had a mean CBS of 19.7 while those who did not receive PBT had a mean CBS score of 17.3 (p=0.11). Patients less than 21 years of age were significantly more likely to have a higher CBS score than those patients 21-64 or >64 years of age (33.5 vs 12.0 vs 14.4, p<0.001). Women were also more likely to have a higher CBS score (23.2 vs 15.1, p<0.001) as were African Americans (19.4) and other races (25.6) when compared to Caucasians (17.3, p=0.0012). Patients who were receiving re-irradiation had a significantly higher CBS than those receiving radiotherapy for the first time (27.8 vs 17.4, p<0.001). Finally, patients insured by Medicare had a significantly higher CBS than those insured by other providers (14.5 vs 10.3, p<0.001). Detailed descriptive statistics are summarized in Table 2.

**Table 2:** Characteristics of patients considered potential candidates for proton beam therapy

Characteristic	n	%	Clinical Benefit Score (CBS)		P Value
			Mean	SD	
Total	205	100	18.7	10.7	
Clinical Benefit Score					<0.001
≤ Median Score	112	54.6	10.4	5.9	
> Median Score	93	45.4	28.8	5.0	
Proton Therapy					0.11
Yes	122	59.5	19.7	10.7	
No	83	40.5	17.3	10.6	
Age (Categorical)					<0.001
<21 Years	12	5.9	33.5	3.3	
21-64 Years	117	57.1	12.0	9.7	
>64 Years	76	37.1	14.4	10.3	
Sex					<0.001
Male	113	55.1	15.1	11.5	
Female	92	44.9	23.2	23.0	
Race					0.0012
Caucasian	138	67.3	17.3	10.8	
African American	42	20.5	19.4	18.0	
Other	25	12.2	25.6	28.0	
Retreatment					<0.001
Yes	27	13.2	27.8	8.5	
No	177	86.8	17.4	10.4	
Insurance					<0.001
Medicare	64	31.2	14.5	10.3	
Other	141	68.8	10.3	10.3	
Site					<0.001
Male Genitourinary	53	25.9	5.1	2.9	
Breast	25	12.2	23.2	6.8	
Central Nervous System	64	31.2	22.5	8.2	
Head and Neck	27	13.2	23.7	8.7	
Thoracic	21	10.2	27.5	6.9	
Other	15	7.3	22.3	7.7	

Multivariate analysis, adjusted for Medicare insurance status, showed that both CBS and insurance status were significant predictors for the receipt of PBT. A one-unit increase in CBS was associated with 1.04 times increased odds of receiving PBT (OR=1.04, 95%CI-1.01-1.07, p=0.0145) while having Medicare was associated with 3.13 times higher odds of receiving PBT (OR=3.13, 95%CI=1.57-6.26, p=0.0012). These results are summarized in Table 3.

**Table 3:** Odds of receiving proton beam therapy during the study period (in the setting of two open treatment rooms) Abbreviation: OR = odds ratio; CI = confidence interval

Characteristic	N	% Receiving Proton Therapy	Unadjusted OR (95% CI)	P-value	Adjusted OR (95% CI)	P-value
Total	205	40.5				
Clinical Benefit Score (Per 1 unit increase)			1.02 (0.99-1.05)	0.11	1.04 (1.01-1.07)	0.0145
Medicare						0.0012
No	141	53.2	Reference		Reference	
Yes	64	73.4	2.43 (1.28-4.64)	<0.01	3.13 (1.57-6.26)	

Subgroup analysis showed similar results when applied to patients who were assessed for radiotherapy in a single gantry setting (prior to April 29, 2016). Multivariate analysis adjusting for insurance status showed, once again, that both CBS and insurance status were significant predictors for the receipt of PBT. A one-unit increase in CBS was associated with 1.05 times increased odds of receiving PBT (OR=1.05, 95%CI-1.00-1.10, p=0.03) while having Medicare was associated with 2.87 times increased odds of receiving PBT (OR=2.87, 95%CI=1.04-7.92, p=0.04). These results are summarized in Table 4.

**Table 4:** Odds of receiving proton beam therapy during the study period (in the setting of one open treatment room) Abbreviation: OR = odds ratio; CI = confidence interval

Characteristic	N	% Receiving Proton Therapy	Unadjusted OR (95% CI)	P-value	Adjusted OR (95% CI)	P-value
Total	84	58.3				
Clinical Benefit Score (Per 1 unit increase)			1.04 (0.99-1.08)	0.08	1.05 (1.00-1.10)	0.03
Medicare						0.04
No	54	51.8	Reference		Reference	
Yes	30	70.0	2.17 (0.84-5.58)	0.11	2.87 (1.04-7.92)	

## Discussion

The CBS was developed within an AMC-RO network in a cost-neutral setting as an allocation tool to address what was anticipated to be a scarce medical resource: the use of PBT during the ramp-up phase of a PC. All patients thought to be candidates for PBT were presented during weekly rounds discussions and were then scored as a means of deciding which patients would be more likely to benefit from PBT. Our study showed that in one and two gantry settings, patients with a higher CBS were statistically more likely to receive PBT, as were those with Medicare health insurance.

Similar resource allocation algorithms have been utilized previously to assign PBT treatment slots [9,11]. Of particular interest is the “proton priority system” (PROPS), which was developed by Bekelman et al. This score comprised components similar to those found in the CBS and included tumor diagnosis, site, stage, performance status, age, urgency of treatment, and clinical trial enrollment status. In a well-designed study, the authors similarly attempted to show an association between the PROPS score and the receipt of PBT using logistic regression. However, they did not find a significant association. They did, however, find a statistically significant association between Medicare insurance status and receipt of PBT [9].

Although the CBS and PROPS are similar, significant differences exist, which may explain why the CBS was statistically associated with the receipt of PBT while PROPS was not. Most importantly, these two scoring systems were utilized in different settings. The CBS was utilized in a setting where only one or two treatment gantries were available for clinical use. Therefore, the demand for PBT was higher than the supply of treatment slots. The Bekelman study was conducted in a setting where all five treatment gantries were available for clinical use. In their paper, they clearly state that during the three-year study period, “our Center’s capacity to provide treatment remained greater than patient demand” [9]. In this setting, it is much more likely that the factors that predict for receipt of PBT would be financial in nature (ex: insurance status).

Another inconsistency between the current study and that of Bekelman et al. is the type of technology that was available. At the time of the PROPS study, only three out of five treatment gantries were utilizing pencil beam scanning technology (PBS), whereas our PC has exclusively utilized PBS in all treatment rooms since opening. PBS delivers more targeted treatment than passively or actively scattered systems, which may allow for the treatment of a broader variety of malignancies. More importantly, this setting, under which the CBS was developed, is more representative of newer PBT facilities currently being built or in the planning stage, which are utilizing PBS technology almost exclusively.

Thirdly, our PC operates in an exclusively “cost-neutral” payer environment, where charges for PBT are similar to those for conventional photon radiotherapy. Our study setting, therefore, adjusts for potential unmeasured confounding, which could occur that would favor allocation towards receiving the cheaper treatment option (photon radiotherapy) despite the patient being more likely to benefit from PBT. The analysis by Bekelman et al. was conducted in a hybrid setting where certain patients were treated using normal PBT pricing and others were treated under “reference pricing,” which is similar to the cost-neutral scenario in our study [12].

Finally, PROPS gives a different weighting to each component of the score [9], whereas CBS gives equal weighting to each component. Although it is difficult to say which approach is superior, it can be argued that CBS is simpler to utilize and, therefore, much easier to implement in a practical setting. There is also less chance of making calculation errors.

Interestingly, both studies found a statistically significant and strong association between Medicare insurance status and the receipt of PBT. Patients in Bekelman’s study were almost two times as likely to receive PBT if they had Medicare as compared to commercial payer insurance or self-payer status and about four times more likely to receive PBT therapy than those with Medicaid [9]. The current study found that patients were about three times more likely to receive PBT if they had Medicare insurance as compared to other forms of insurance. These findings support the strong association between Medicare insurance status and receipt of PBT.

Similar decision-making algorithms have been utilized in the non-oncologic setting previously and include the model for end-stage liver disease (MELD) score used to prioritize patients for liver transplantation or other scores that prioritize patients who are likely to benefit from admission to intensive care units [13-16]. These tools are available to assist physicians in the decision-making process and can serve a useful purpose within a multi-faceted decision-making process. The CBS is similar in this regard and can be used to guide the decisions of not only radiation oncologists but also of referring primary care providers, oncologists, surgeons, and nurses.

The limitations of the current study include its single institutional nature. It must also be acknowledged that the CBS was developed based on the expert opinion and scientific literature was available at the time [4-5,8]. Although the state of knowledge has evolved since, there continues to be a lack of high-level evidence comparing PBT and photon therapy [17]. However, a number of trials are ongoing to address this issue in several disease sites [18-23]. The current study should not be used to discount or downplay the importance of comparative, phase three, randomized controlled trials but should only aim to supplement their results.

The CBS has not yet been validated independently and further comparative effectiveness research is required to strengthen its use in a clinical setting. As more high-level evidence becomes available, CBS should be reanalyzed in settings of proven survival advantage and decreased toxicity. Finally, most operating PBT centers in the United States and many around the world do not operate in cost-neutral payer settings, which could theoretically limit the generalizability of our study results. Further validation of the CBS will be needed in non-cost-neutral settings.

## Conclusions

In conclusion, at our center, the CBS was implemented to aid with PBT resource allocation and the system was well adhered to with higher CBS scores significantly predicting for receipt of PBT. Radiation oncologists working in, or other health care providers referring to, one- or two-room PBT facilities, may consider utilizing the CBS to prioritize patients who are more likely to benefit from the receipt of PBT. The CBS can also be used in larger PBT facilities where resource allocation may be an issue. Further validation of the CBS is needed and its use is sure to evolve as results from currently ongoing comparative effectiveness trials comparing PBT to photon therapy become available.
